# *Epigenetic Editing*: targeted rewriting of epigenetic marks to modulate expression of selected target genes

**DOI:** 10.1093/nar/gks863

**Published:** 2012-09-21

**Authors:** Marloes L. de Groote, Pernette J. Verschure, Marianne G. Rots

**Affiliations:** ^1^Department of Pathology and Medical Biology, University Medical Center Groningen, University of Groningen, Hanzeplein 1 EA11, 9713 GZ, Groningen and ^2^Swammerdam Institute for Life Sciences, The Netherlands Institute for Systems Biology, University of Amsterdam, Science Park 904, 1098 XH Amsterdam, The Netherlands

## Abstract

Despite significant advances made in epigenetic research in recent decades, many questions remain unresolved, especially concerning cause and consequence of epigenetic marks with respect to gene expression modulation (GEM). Technologies allowing the targeting of epigenetic enzymes to predetermined DNA sequences are uniquely suited to answer such questions and could provide potent (bio)medical tools. Toward the goal of *gene-specific* GEM by overwriting epigenetic marks (*Epigenetic Editing, EGE*), instructive epigenetic marks need to be identified and their writers/erasers should then be fused to gene-specific DNA binding domains. The appropriate epigenetic mark(s) to change in order to efficiently modulate gene expression might have to be validated for any given chromatin context and should be (mitotically) stable. Various insights in such issues have been obtained by *sequence-specific* targeting of epigenetic enzymes, as is presented in this review. Features of such studies provide critical aspects for further improving *EGE*. An example of this is the direct effect of the edited mark versus the indirect effect of recruited secondary proteins by targeting epigenetic enzymes (or their domains). Proof-of-concept of expression modulation of an endogenous target gene is emerging from the few *EGE* studies reported. Apart from its promise in correcting disease-associated epi-mutations, *EGE* represents a powerful tool to address fundamental epigenetic questions.

## INTRODUCTION

**Figure 1.**
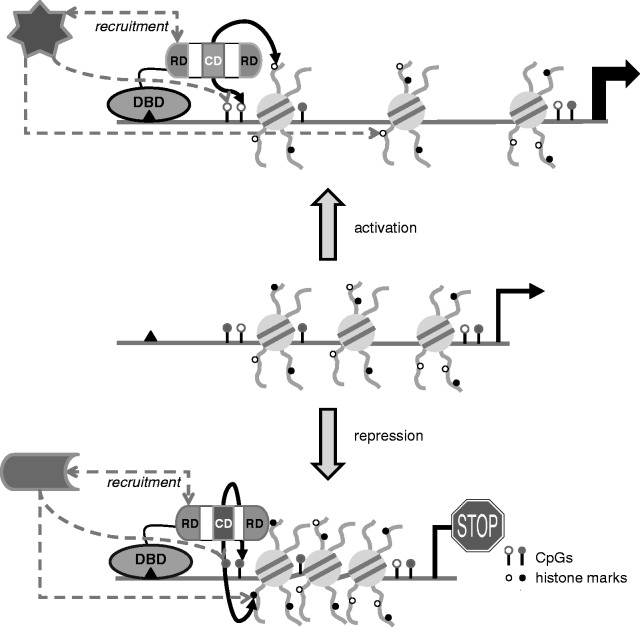
Targeted rewriting of epigenetic marks. Schematic figure shows the general concept of targeting epigenetic enzymes. In the middle, an example of a certain locus harboring a DBD recognition site (black triangle) is shown. Lollypops represent either unmethylated (open) or methylated (filled) CpGs. Histones and their tails are also represented. Histone tails can be post-translationally modified and as such are associated with a repressed chromatin state (represented by the filled black dots), or with an active chromatin state (represented by open black circles). The upper and lower figures show the induced change in gene expression by targeting a DBD fused to an epigenetic enzyme involved in changing the epigenetic composition (histone modifications or DNA methylation), thereby causing gene activation (top) and repression (bottom). In the epigenetic enzymes, CD = catalytic domains and RD = recruiting domains are indicated. Black arrows show the action of the CD of the epigenetic enzymes, dashed arrows show the possible recruitment of other proteins or capturing by other proteins (star, top; shape, bottom).

Epigenetics is gaining momentum in nearly all biomedical research fields, and substantial knowledge of the epigenetic marks (e.g. DNA methylation and post-translational histone modifications) and enzymes involved in reading, writing and erasing of such marks have been obtained (1–5). The majority of the data reported so far, however, does not provide insights in, e.g., the relative importance of the marks with regard to gene expression control, nor in the order of events. In general, the performed studies tend to be descriptive and epigenetic questions related to cause or consequence effects of epigenetic marks with respect to gene expression modulation (GEM) are under debate (6–8). In light of the fact that such fundamental questions have only been addressed in a few studies, including (9–11), targeting epigenetic enzymes to particular chromatin landscapes will provide useful insights. This review sets out to summarize the outcome of targeting approaches with respect to the effect of epigenetic writers and erasers on the chromatin state and/or on gene expression (Figure 1 and Tables 1–3).

**Table 1.** Targeted DNA methylation editors

Enzyme	DBD	Target	EGE	GEM	References
M.SssI	ZF	Oligo, endogenous target (yeast)	✓	n.a.	(48,49)
	TFO	Plasmid DNA (cell free)	✓	n.a.	(50)
M.HhaI	ZF	Oligo (cell free)	✓	n.a.	(51–53)
		Plasmids in bacteria	✓	n.a.	(52,53)
		Integrated (bacteria)	✗	n.a.	(52)
M.HpaII	ZF	Oligo, plasmid DNA (cell free)	✓	n.a.	(51,52)
		Plasmids/integrated (bacteria)	✓	n.a.	(52)
		Integrated (mammalian)	✓	↓	(52,54)
mDnmt3a FL	Gal4	Reporter plasmid (mammalian)	✗	↓	(30)
mDnmt3a CD	Gal4	Reporter plasmid (mammalian)	✓	↓	(55)
	ZF	Reporter plasmid (mammalian)	✓	↓	(55)
	ZF	Viral DNA	✓	n.a.	(55)
hDnmt3a CD	ZF	Mitochondrial DNA	✓	n.a.	(56)
		Endogenous target (mammalian)	✓	↓	(57)
mDnmt3b CD	Gal4	Reporter plasmid	✓	↓	(55)
Tet1	Gal4	Integrated (mammalian)	n.a.	↓	(58)
5-MCDG	RXRα-receptor	Integrated (mammalian)	✓	↑	(59)
	LexA	Integrated (mammalian)	n.a.	✗	(60)
TDG	NFκB DBD	Endogenous targets (mammalian)	✓	↑	(21)
VP64	ZF	Endogenous target (mammalian)	✓	↑	(61)

✗, no effect; n.a., not assessed; ✓, effect reported; ↓, downregulation; ↑, upregulation.

**Table 2.** Targeted repressive histone modifying enzymes

Enzyme	Aka (68)	DBD	Target	EGE	GEM	References
HDAC1		Gal4	Reporter plasmid (in vial)	n.a.	✓	(69)
			Reporter plasmid	n.a.	✓	(70,71)
HDAC2/RPD3		Gal4	Reporter plasmid	n.a.	✓	(71,72)
		LexA	Reporter plasmid	n.a.	✓	(73,74)
HDAC3		Gal4	Reporter plasmid	n.a.	✓	(71)
Sirt1		Gal4	Reporter plasmid	n.a.	✓	(75)
			Integrated (mammalian)	✓	✓	(75)
LSD1		Gal4	Integrated (mammalian)	✗	n.a.	(76)
		TetR	Integrated (artificial chromosome)	✓	✓	(77)
Setdb1	KMT1E	Gal4	Reporter plasmid	✓	✓	(30)
G9a	KMT1C	Gal4	Integrated (mammalian)	✓	✓	(78)
		ZF	Endogenous target	✓	✓	(29)
Suv39H1/SU(VAR)3-9	KMT1A	Gal4	Reporter plasmid	n.a.	✓	(79,80)
		ZF	Endogenous target	✓	✓	(29)
Ezh2	KMT6	Gal4	Integrated (mammalian)	✓	✓	(81)
				✓	n.a.	(76)
vSet		Gal4	Reporter plasmid	n.a.	✓	(82)
Set2	KMT3A	LexA	Reporter plasmid	n.a.	✓	(73)
Smyd2	KMT3C	Gal4	Reporter plasmid	n.a.	✓	(83)

Aka, also known as; ✗, no effect; n.a., not assessed; ✓, effect reported.

**Table 3.** Targeted activating histone modifying enzymes

Enzyme	Aka (68)	DBD	Target	EGE	GEM	References
p300	KAT3B	Gal4	Reporter plasmid	n.a.	✓	(98–101)
			Reporter plasmid	✓	✓	(31)
		LexA	Integrated (mammalian)	n.a.	✓	(60)
		MBD	Endogenous targets	n.a.	✓	(18)
PCAF	KAT2B	Gal4	Reporter plasmid	n.a.	✓	(100)
		LexA	Integrated (mammalian)	n.a.	✓	(60)
		MLL	Endogenous targets	n.a.	✓	(20)
CBP	KAT3A	Gal4	Reporter plasmid	n.a.	✓	(102–106)
		MLL	Endogenous targets	n.a.	✓	(20,107)
GCN5	KAT2A	MLL	Endogenous targets	n.a.	✓	(20)
Meisetz		Gal4	Reporter plasmid	n.a.	✓	(108)
Ash1	KMT2H	Gal4	Integrated (drosophila)	✓	✓	(109)
		LexA	Integrated (mammalian)	n.a.	✗	(60)
Dot1/Dot1L	KMT4	MLL	Endogenous targets	✓	n.a.	(19)
		LexA	Integrated (yeast)	n.a.	✓	(110)
JMJD2D	KDM4D	MBD	Endogenous targets	✓	✗	(17)
KIAA1718	KDM7A	Gal4	Integrated (mammalian)	✓	✓	(111)

Aka, also known as; ✗, no effect; n.a., not assessed; ✓, effect reported.

The general principle of DNA-sequence-specific targeting systems is the fusion of (a part of) an epigenetic enzyme to a DNA binding domain (DBD) to enforce the presence of this effector domain on a particular DNA sequence (Figure 1). This target DNA sequence is often located within an oligonucleotide, a plasmid or integrated in a particular chromatin environment in the genome of a cell. In addition to the induced epigenetic changes, the effect of targeting epigenetic enzymes on gene expression can be assessed by measuring gene expression levels of (reporter) genes that lie in close proximity of the DBD recognition site. Most of the reported targeting efforts make use of non-mammalian DBDs and (multiple repeats of) their specific recognition sequences including the yeast Gal4-UAS system (12), the prokaryotic Tet Repressor (TetR)-Tet Operator (TetO) (13), Lac Repressor (LacR)-Lac Operator (LacO) (14,15) and the LexA repressor-LexA operator system (16). Additionally, some mammalian systems have been exploited to target enzymes to native endogenous chromatin sites, like the Methyl Binding Domain (MBD) of MeCP2 to target enzymes to genomic sites consisting of hypermethylated DNA (17,18), the DBD of Mixed Lineage Leukemia (MLL) to force epigenetic enzymes to reside on endogenous MLL target genes (19,20) and the DBD of NFκB to affect NFκB targets (21).

All of the above mentioned systems, however, are somewhat limited with regard to addressing biologically relevant questions, because of the need to introduce foreign DBD recognition sites in the host cells, or because they are not specifically targeting one unique site in the genome, but multiple endogenous target sites of the DBD. To specifically bind one genomic address, as explored by Artificial Transcription Factors (ATFs) (22), various classes of DBDs can be engineered, such as designer zinc finger (ZF) proteins (23), Triplex Forming Oligos (TFOs) (24) and the recently described TALE domains (25). Indeed, with the developments in the field of genome editing [where nucleases are fused to sequence-specific DBD proteins to introduce site-specific DNA cleavage: Methods of the Year 2011 (26)], the targeting of epigenetic editors (writers or erasers) to a specific gene has come within easy reach.

The effective binding of the (gene-specific) DBDs to various euchromatin and heterochromatin targets has been shown by fusing the DBDs to transcription activating or repressive domains (VP64 or SKD, respectively), which recruit other proteins to induce (27) or repress (23) target gene expression. Despite their success (22,23,28), ATFs are likely to function only transiently and active gene-specific overwriting of epigenetic marks by targeting epigenetic enzymes or domains thereof (*Epigenetic Editing, EGE*) might provide advantages with respect to long-term modulation of gene expression. Apart from obtaining sustained GEM, other advantages of *EGE* include upregulation by allowing natural expression mechanisms to occur, as opposed to mere overexpression as obtained by gene therapy or ATFs. Moreover, the approach of *EGE* is uniquely suited to investigate functions of epigenetic writers and erasers and to elucidate consequences of epigenetic marks at any given chromatin environment, providing insights in gene expression regulation mechanisms. In this review, an overview of studies on *sequence-specific* and the few *gene-specific* targeted epigenetic editors will be presented. Also, some points from these studies will be highlighted that will further improve gene-specific *EGE* efforts. Although essential information on chromatin behavior has been obtained by targeting epigenetic readers as well, this review will only focus on direct modulation of epigenetic marks by the implicit targeting of writers and erasers.

## GENE EXPRESSION MODULATION WITH TARGETED EPIGENETIC EDITORS

A substantial amount of studies on targeting epigenetic enzymes has provided strong indications that epigenetic editors can be targeted to obtain a change in gene expression. These studies, as discussed in this review, have been using diverse experimental designs ranging from oligonucleotides to endogenous genes in the natural chromatin context. Thus far, the observed effects of targeted epigenetic enzymes (including histone modifying enzymes) have been mainly obtained by co-transfection experiments. Such experiments more closely resemble the endogenous situation than test-tube oligonucleotides experiments, since the fusion proteins (and their target plasmid) encounter endogenous factors that might play a role, or are even required, in changing essential epigenetic marks and/or in obtaining GEM. However, the use of cotransfections to analyse the effects of targeted histone modifiers is subject of debate. Whereas it has been reported that plasmids are not suitable for establishing the effects of targeting histone modifying enzymes because of the lack of chromatinization (29), others show by ChIP that a molecular effect of targeting histone modifying enzymes can be observed, indicating that histones can get associated with the plasmid (30,31). These latter studies confirm nuclease-digestion experiments demonstrating the association of nucleosomes on plasmid DNA (32–34). More informative data, however, can be derived from targeting epigenetic editors to exogenous sites (usually including a reporter gene) integrated in the chromatin context of cells. Although this approach is also artificial and integrated sites are more susceptible to epigenetic silencing, it provides insights in the effect of induced changes on the chromatin context and is suitable to address heritability issues. True *EGE*, where a single endogenous gene is targeted, obviously provides the most relevant information and the few studies published so far will be discussed in more detail.

As described below, although not all of the targeting studies describe effects on both molecular epigenetic level and GEM, some (including the *EGE* studies) clearly indicate the causal relationship between the rewritten mark and GEM. Because of differences in experimental design (such as the type of DBD or design and expression of the construct) it is difficult to compare efficacy of targeted GEM between studies. It is tempting to speculate that an absence of effect on gene expression upon inducing a change in one epigenetic mark can be explained by the native chromatin context, as exemplified by the protection of DNA for CpG methylation when histone 3 lysine 4 is methylated, which thus might prevent spreading (35–37). Other marks that remain present in the chromatin context of the targeted gene might recruit enzymes to restore the initial epigenetic profile. In this regard, it is currently unclear if more than one mark needs to be changed to facilitate an effective change in gene expression in the endogenous situation. Furthermore, the change of more than one epigenetic mark could very well be required for heritability of the effect, which would be of importance for therapeutic approaches in particular. Nevertheless, as described in this first part, promising results have been obtained by the active change of just one epigenetic mark and approaches to further improve *EGE* arising from such studies will be discussed in the second part.

### Downregulation of gene expression via rewriting of epigenetic marks

In general, targeted epigenetic silencing has advantages over siRNA approaches, which are currently widely exploited for various clinical phenotypes, as reviewed in (38), but which are generally transient and suffer from target-independent effects (39,40). An alternative (synergistic) approach to downregulate the expression of a gene of interest is by ATFs, which might proof efficient as only two copies of DNA need to be targeted in every cell, as opposed to numerous continuously produced mRNA molecules. Although significant repression has been achieved for various endogenous genes by fusions of ZFs to the KRAB domain (23,28), the KRAB domain does not have enzymatic activities by itself and therefore does not *directly* interfere with the epigenetic context at the target site. In this respect, direct targeting of epigenetic enzymes to endogenous target sequences (*EGE*) is more relevant, both for biological questions as well as for the potential use of *EGE* as a therapeutic approach in the future. In this section, studies on repressive effector domains targeted to actively interfere with epigenetic marks in order to repress gene expression are discussed.

#### Targeted DNA methyltransferases

DNA hypermethylation, especially around the transcription start site and exon 1 (41,42), is strongly associated with inactive genes. Moreover, DNA methylation is in principle faithfully inherited during mitosis, and has been reported to serve as a strong molecular mark for gene silencing memory (43–45). Therefore, to permanently downregulate the expression of a gene, targeting DNA methyltransferases (MTases) is an obvious choice. Some alternative gene-specific technologies to induce DNA methylation have been described, including RNA-directed (46) and methylated oligo-induced (47) methylation, but the general applicability of such approaches to silence any gene of interest is unclear. Nowadays, DBDs can be engineered to specifically bind virtually any gene (22,23) to target transcriptional repressors to these genes, subsequently decreasing gene expression. Indeed, upon fusion of such engineerable DBDs to the KRAB domain, effective reduction of oncogene expression (resulting in reduced tumorigenicity) was shown (28). Thus, fusion of DNA MTases to such domains offers an appealing approach for inducing inheritable gene silencing. In fact, DNA MTases have been extensively studied in fusions to ZFs (Table 1), as also reviewed in (62).

Indeed, upon targeting by fusion to gene-specific (ZFs or TFOs) or sequence-specific (Gal4) DBDs, both the prokaryotic DNA MTases M.SssI, M.HhaI and M.HpaII as well as the catalytic domains (CDs) of the mammalian enzymes mDnmt3a and mDnmt3b showed efficient preferential DNA methylation of target sites in oligonucleotides (48,50–53) or on reporter plasmids (30,50,52,53,55) and when assessed, the targeted DNA methylation upon cotransfections was correlated to repression of reporter gene expression (Table 1). The ability of a ZF fused to a prokaryotic DNA MTase to cause preferential DNA methylation at an endogenous mammalian target site was observed for M.SssI in the context of yeast chromatin (49). Noteworthy, the ZF binding site itself was not methylated, indicating protection from direct DNA methylation by the ZF binding. As yeast cells have no endogenous DNA methylation system, targeting specificity can be easily investigated. In this respect, this yeast study—as confirmed in some of the other studies (48,49,52)—revealed additional aspecific background DNA methylation, which will be further discussed in *‘TOWARD* SPECIFIC *GENE EXPRESSION MODULATION—The effector domain’.*

To increase specificity of the targeted DNA methylation by prokaryotic enzymes, ZFs were fused to less active mutants of these prokaryotic DNA MTases (M.HhaI^Q237G^ and M.HpaII^F35H^ (52). In contrast to ZF-M.HhaI^Q237G^, ZF-M.HpaII^F35H^ was able to induce DNA methylation on its target site, integrated in the bacterial genome. Moreover, when targeting a site integrated in the mammalian genome, ZF-M.HpaII^F35H^ could induce targeted DNA methylation as well as downregulation of the reporter gene (54). Interestingly, the histone modification state of the ZF target site accordingly changed into a repressive state, as an enrichment of H3K9me2 and a reduction of H3K4me3 was observed at the site of the integrated reporter gene where DNA methylation was induced (54). This indicates that active change of one epigenetic mark (in this case DNA methylation) can cause a cascade of changes, which might reinforce the repressive state of the chromatin at the target gene. Such a reinforced repressive chromatin state could explain the observation of remaining DNA methylation at the target site after several cell passages. Moreover, the stable DNA methylation state was associated with stable reporter gene repression at least up to 17 days after the expression of ZF-M.HpaII^F35H^ was no longer detected at both the RNA and protein level (at 6 or 7 days after transfection, respectively) (54). This sustained DNA methylation and repression of gene expression is indicative of the DNA methylation induced by ZF-M.HpaII^F35H^ being inherited through cell divisions. This study thus provides proof-of-concept that targeted DNA methylation can be exploited for sustained gene repression.

Compared with prokaryotic MTases, mammalian DNA MTases, i.e. Dnmt1, 3a and 3b, display several advantages when considered for use in targeted DNA methylation of the mammalian genome: although, like M.SssI, mammalian DNA MTases can methylate all CpG sequences without any further sequence-restrictions, the low catalytic activity of the mammalian enzymes (63) might better allow restriction of methylation to targeted CpGs by fusion to DBDs. In addition, since being mammalian, the MTase activity is probably not influenced by DNA-histone interactions that theoretically might hamper the prokaryotic DNA MTases, as these originate from histone-less organisms (64). Moreover, mammalian enzymes are more likely to recruit other mammalian proteins important for reinforcement of transcriptional repression. This is nicely exemplified by the Gal4-mDnmt3a full-length cotransfection study (Table 1): although no detectable DNA methylation was induced at the target region by this construct, repression of reporter gene expression was observed (30). Recruitment of endogenous co-factors has been observed for this and several other epigenetic effector domains and will be discussed later. Last but not least, the domain will evoke less immunogenicity since it is less foreign to the organism.

The ability to cause preferential DNA methylation at a cellular target site was observed for a ZF fused to hDnmt3a CD, targeting mitochondrial DNA (56). In this study, 23% of the clones analysed by bisulfite sequencing showed preferential methylation at the cytosine directly adjacent to the ZF binding site (56). Another interesting parameter that has been addressed in this study is spreading of the epigenetic mark, which was observed within a region of at least 120 bp surrounding the ZF target site (56). In another study, where DNA methylation was targeted to successfully methylate viral DNA upon cellular infection, spreading up to 380 bp on either site of the DBD recognition site has been observed (55). However, it is not directly clear whether the observed induction of distant DNA methylation in these studies is truly because of spreading or because of the flexibility of the targeting construct or the target DNA.

Only very recently, the first *gene*-specific ZF-targeted DNA methylation was reported in the nuclear chromatin context for the tumor suppressor gene MASPIN and the oncogene SOX2 (57). Upon targeting the CD of Dnmt3a to the promoter of the MASPIN gene, pronounced targeted DNA methylation (of 50%) occurred for two target CpGs. In addition, differential positioning of induced DNA methylation was obtained by this EGE approach. The targeted DNA methylation was sufficient to efficiently downregulate MASPIN expression, with up to 90% repression observed in single clones. Efficient repression without dense DNA methylation is in line with other observations, like for p53 where induced DNA methylation of a single-specific CpG was shown to severely decrease gene expression (65). Interestingly, the observed MASPIN downregulation was stably inherited: up to 50 days post-infection, when the expression of the ZF-Dnmt3a CD fusion was barely detectable, gene expression was still repressed. Moreover, treating cells at this time point with the DNA methylation inhibitor 5-azadeoxycytidine released the repression, indicating that the induced DNA methylation is still present. Indeed, methylation patterns remained similar to the patterns observed soon after transductions. Furthermore, knockdown of UHRF1 expression, a protein involved in DNA methylation maintenance, caused significant re-expression of MASPIN. The findings were extended to the oncogene SOX2, which could also be efficiently repressed upon targeting Dnmt3a CD to its promoter by fusion to another ZF. By making use of a doxycycline inducible promoter, expression of the ZF-Dnmt3a CD could be cleared after 48 h, allowing the cells to recover from repression. Interestingly, the ZF-Dnmt3a cells did not recover cell proliferation, while cells conditionally expressing a ZF-KRAB derivative did recover. Studies like these indicate the benefit of using *EGE* over targeting the KRAB domain.

#### Targeted repressive histone modifying enzymes

As an alternative (or synergistic) approach to the introduction of DNA methylation at transcription start sites, repression of gene expression can be achieved by targeted modification of histone tail residues. In this respect, repressive chromatin covered by histone H3K9 methylation, exhibits a chromatin state that is proposed to be able to spread its epigenetic composition for instance via Heterochromatin Protein 1 (HP1)-induced heterochromatinization (66,67). As such, H3K9 MTases (such as Setdb1, G9a and Suv39H1/SU(VAR)3-9) are of interest to fuse to DBDs for repression of target gene expression. Furthermore, histone H3K27 methylation represents a chromatin state related to Polycomb group protein (PcG) regulated genes that become stably silenced during differentiation and cell-fate determination, which makes H3K27 MTases (like Ezh2 or vSet) other interesting candidates for targeted repression of gene expression. Indeed, H3K9, H3K27 and H3K36 MTases (Set2 and Smyd2) as well as an H3K4 demethylase (LSD1) have been targeted leading to gene repression in all cases where gene expression was assessed (Table 2).

Interestingly, lysine residues like H3K9 and H3K27 can be either acetylated or methylated, and deacetylation of acetylated histone lysine residues is required before the induction of histone methylation on these lysine residues can take place (84). It has long been thought that because acetylation neutralizes the positive charge on the histones, this modification facilitates the open configuration of the chromatin at actively expressed regions in the chromatin (85,86). Indeed, hypoacetylation is found at promoter sites of genes with low or no expression levels, whereas acetylated histone tails are mainly associated with active genes (84,87). In addition to this ‘charge hypothesis’, acetylation also recruits activating protein complexes (including chromatin ‘readers’) by changing the histone modification composition of the chromatin (88,89). Thus, to silence genes through *EGE*, also HDACs are among the candidate enzymes to be targeted. HDACs of class I (HDAC1, 2 and 3) as well as the sirtuin SirT1 (NAD+ dependent, class III) have been targeted in cotransfection studies using the Gal4 or LexA DBD and indeed reduced gene expression (Table 2).

Although promising, most studies were not intended to assess the actual induction of the histone mark and no conclusions can be drawn from these studies regarding causal relationships between histone marks and gene expression. In contrast, the anticipated targeted change of histone modifications was assessed and reported for Setdb1 (30) upon cotransfections and for Sirt1 (75), LSD1 (77), G9a (78) and Ezh2 (76,81) targeted to integrated target sites (Table 2). Accordingly, these changes in epigenetic marks were associated with repression of gene expression.

Interestingly, targeting of Gal4 fused to Sirt1 (75), G9a (78) and Ezh2 (81) to integrated target sites, caused other changes in histone marks in addition to the ones anticipated, as also described for targeted DNA methylation. This again might indicate that the change of one mark can induce a cascade of changes in chromatin modifications which probably reinforces the repressed state and might also add to the persistence of repression. Indeed, mitotically inherited repression of target gene expression was noted at least up to 4 days after clearance of the tetracycline-inducible expression of the Gal4-Ezh2 fusion protein in human cells (81), associated with both the anticipated H3K27 methylation and additional H3K4 demethylation. In a murine study, Gal4-Ezh2 did not change other marks than H3K27 methylation (76). In fact, although targeting Ezh2 was shown to recruit Dnmt3a to the integrated target site, no DNA methylation was observed and permissive chromatin marks (H3K4me2, H3Ac) remained present. The absence of DNA methylation, despite the presence of Dnmt3a, might be explained by the presence of H3K4 methylation, as this mark seems to prevent DNA methylation (35–37) and as such might need to be removed by a specific histone demethylase before repression of gene expression can take place. In this respect, targeting of TetR-LSD1 to an artificial chromosome resulted in demethylation of H3K4me3 (without affecting H3K9/K27 methylation) and induction of gene expression (77). However, in another study, targeting of Gal4-LSD1 to a target integrated in mammalian cells, with the aim to allow DNA methylation to be induced upon targeting of Ezh2, was not successful (76). It might be that this discrepancy is caused by the chromatin context of the artificial chromosome where LSD1 was targeted to by fusion to TetR, which made it easier to reach the target or to affect the histone modification levels. However, since the experimental designs were so different, it is difficult to compare the two studies.

The crosstalk between histones and DNA methylation (90,91) has also been described for other histone marks than H3K4 methylation. In this respect, the Histone Methyl Transferase (HMT) G9a, which induces H3K9 methylation, HP1 binding, local heterochromatin formation and gene silencing, can also recruit DNA MTases Dnmt3a and 3b which catalyse *de novo* DNA methylation (92,93). Similarly, in addition to loss of H3K9 methylation at major centromeric satellites in Suv39h knock-out embryonic stem cells, also a decrease in Dnmt3b dependent CpG methylation has been observed (94). Targeting of such writers might thus result in efficient repression of gene expression. Indeed, gene-specific ZF-targeted histone modifications result in repression of a target gene in the endogenous chromatin context by targeting G9a/Suv39H1 or a histone deimination domain (29,95). Targeting of G9a or Suv39H1 by fusion to a three-finger ZF designed to bind the gene of interest (VEGF-A), provides the first example of ZF-mediated *EGE* of an endogenous gene (29). This VEGF-A ZF, when fused to a transcriptional activation domain like Viral Protein 16 (VP16) of HSV (Herpes simplex virus type 1), caused upregulation of endogenous VEGF-A expression (96) and has been further investigated in phase II clinical trials after fusion to the activator p65. Despite a lack of improved therapeutic effect over placebo treatment (97), these efforts demonstrate the feasibility of targeting genes in a clinical setting by ZFs. Swapping the transcriptional activation domain with the catalytic C-terminal domain of H3K9 MTase G9a (N-terminal 828 amino acids (aa) removed) or with smaller N-terminal deletions (Δ75 aa or Δ148 aa) of Suv39H1, caused induction of at least H3K9me2 as well as repression of the endogenous target gene (29). The effect of the H3K9 MTases (inducing the anticipated mark and repressing gene expression upon targeting) was validated by us for another endogenous gene by fusing the enzymes to another ZF (Falahi *et al.*, submitted for publication). For VEGF-A, increased levels of H3K9me2 were observed throughout the investigated region, up to 900 bp away from the ZF binding site upon targeting either Suv39H1 (Δ75 aa N-terminal) or the CD of G9a (29). This indicates that the targeting of an H3K9 MTase enables the activation of an endogenous mechanism spreading the H3K9 methylation marks and thereby reinforcing repression. Interestingly, in this study and the one on targeted deimination by Cuthbert *et al.* (95), the targeting construct was delivered to the cells via transient transfection of an expression plasmid, whereas the only other example of true endogenous *EGE* delivered the construct virally (57). Unfortunately, the observed effects were not followed in time, which would have been interesting because prolonged effects would indicate that the targeted induction of the mark is mitotically inherited.

### Induction of gene expression via rewriting of epigenetic marks

Also for induction of gene expression, to reactivate epigenetically silenced genes (for example tumor suppressor genes in cancer), there is a variety of possibilities. The achievements obtained by targeted DNA demethylation, locus-specific addition of acetyl groups to histone tail residues, methylation of H3K4 or H3K79 and demethylation of H3K9 or H3K27 will be discussed in this part (Tables 1 and 3).

#### Targeted ‘DNA demethylases’

To achieve long term re-expression, it seems apparent that the removal of DNA methylation is an important step, at least around the transcription start site and exon 1 of the target gene (41,42). However, until quite recently it was not generally accepted that active DNA demethylation occurs in mammals, even though examples had been described of both global and locus-specific *active* DNA demethylation, as reviewed in (112). In this respect, straightforward mammalian effector domains to obtain targeted DNA demethylation are not available. Now that the concept of active DNA demethylation in mammals is increasingly accepted, efforts to identify mammalian proteins associated with the process of active DNA demethylation resulted in interesting candidates for targeted removal of DNA methylation marks as reviewed in (112,113). In fact, several mechanisms and proteins were described to be associated with DNA demethylation. In plants, enzymes involved in DNA demethylation are relatively well established. Repressor of silencing 1 (Ros1), Demeter (DME) and Demeter-like proteins (DML2, DML3) are unambiguously associated with active DNA demethylation via base excision repair (BER) in plants (114,115). Interestingly, although other epigenetic plant enzymes have been shown to function in a mammalian setting (29), no efforts were reported on expressing or targeting the plant CpG demethylation enzymes in mammalian cells.

Potential mechanisms of active DNA demethylation in mammals, for which some indications have been described, are (i) direct removal of the methyl group; (ii) 5meC glycosylation followed by BER (like in plants); (iii) deamination followed by mismatch repair; and (iv) nucleotide excision repair (112,116). However, most of these mechanisms are still under debate. One mechanism that is now accepted to play a role in DNA demethylation is oxidation of 5-methylcytosine to 5-hydroxymethylcytosine (5hmC) by the Tet enzymes. The biological role of 5hmC itself is not fully known yet. However, the 5hmC mark has been associated with active genes (117), specifically at the promoter (118) but also in the gene bodies (119,120). Similarly, ChIP-seq studies demonstrated Tet1 to be present on genes occupied by H3K4me3 (active), H3K27me3 (inactive) or both (bivalent domains) and promoter activity can not be predicted by Tet1 binding (58). For *EGE* purposes, it is noteworthy to mention that 5hmC has been proposed to be an intermediate in the active DNA demethylation pathway (121). Consistent with this, intermediates that might be formed in the process of converting 5hmC to be eventually replaced by an unmodified cytosine were detected recently (122).

Interestingly, upon targeting of Tet1 to five Gal4-binding sites integrated in mammalian cells, repression of the targeted integrated reporter gene was observed (58). Also recruitment of Sin3a, a protein that is part of a transcriptional repression complex could be detected. Unfortunately, this targeting study did not investigate effects on the DNA methylation status of CpGs in the targeted site. In addition, Tet1 was not targeted to hypermethylated (inactive) genes, so its effects on gene expression in a heterochromatin context are currently largely unknown.

As also suggested in one of the many reviews about DNA demethylation (116), targeting candidate DNA demethylases of the proposed possible pathways to specific genes in different chromatin contexts will provide more insights into the enzyme or enzymes that can truly actively demethylate methylated CpGs. However, only a few studies have employed targeting of potential DNA demethylases so far, of which one not even intentionally: upon overexpression of 5-MCDG, presently known as Thymine DNA Glycosylase (TDG), the protein associated with the retinoid receptor RXRα (59). A reporter transgene was bound by this receptor and (consequently) DNA demethylation and upregulation of the expression of this gene were observed. Interestingly, TDG was recently fused to the DBD of NFκB, which also led to some DNA demethylation of endogenous NFκB target genes and an increase in target gene transcription (21). In contrast, in another study, aiming to prevent silencing, no effect on the expression of an integrated target gene was seen upon stable transfection of LexA-MCDG in mammalian cells (60).

Alternatively, a ZF targeting study in mammalian cells reported on DNA demethylation induced by targeting a transcriptional activator (VP16-tetramer; VP64) to an endogenous gene (61). Effectively, a gene-specific upregulation of gene expression of 25- to 125-fold on mRNA level was achieved, associated with DNA demethylation of up to 70% of the targeted CpGs. Although it might be suggested that the DNA demethylation is a secondary effect of the VP64-induced transcription, the precise location of demethylation, which is strictly determined by the orientation of the effector domain, would argue against this. Still, it needs to be further investigated whether the DNA demethylation is due to active DNA demethylation by VP64 or its recruited proteins, or whether the effect is merely a consequence of steric hindrance by recruited protein complexes preventing Dnmt1 from copying methylation to the daughter strand upon cell division. In plants, transient transfection of gene-specific ZFs fused to VP64 even resulted in heritable (at least two subsequent generations) activation of gene expression of the targeted gene (123). The sustained effect observed here might also be associated with epigenetic changes like DNA demethylation, although this was not addressed in the specific study.

#### Targeted activating histone modifying enzymes

Although DNA methylation provides a powerful silencing memory, it is not necessarily a lock for gene expression and many genes have been found to be upregulated despite their DNA hypermethylation status after treatment of cells with HDAC inhibitors as described in (43) and references therein. Thus, instead of (or in addition to) DNA demethylation, gene-specific removal of repressive histone marks and/or induction of activating histone marks might achieve efficient and lasting upregulation of target gene expression. Histone modifying enzymes that are likely to be of interest for obtaining targeted activation of gene expression are histone acetyltransferases (HATs; such as p300, P/CAF, CBP and GCN5) and histone methyltransferases methylating histone tail residues H3K4 or H3K79 (for instance Meisetz and Ash1 or Dot1/Dot1L, respectively). Several ‘activating’ histone modifying enzymes have been targeted to predetermined target sites (see Table 3 for an overview) within reporter plasmids, integrated within host genomes or to endogenous target sites by using the endogenous DBDs MLL or MBD.

Irrespective of the context of the target gene, upregulation of gene expression was seen for most of the activating histone modifying enzymes that were targeted (p300, P/CAF, CBP, GCN5, Meisetz, Ash1 and Dot1). Since most cotransfection studies intended to examine the role of co-activators, molecular chromatin marks were generally not studied (and if so, to a low extent). Importantly, the one cotransfection study that assessed molecular chromatin marks on plasmid level upon targeting of an activating histone modifying enzyme indeed showed an increase in acetylation by targeted p300 (31).

Interestingly, despite using the same DBD, different genes can be affected when (domains of) other enzymes are fused. Namely, when the HAT domain of CBP in a fusion of CBP to MLL was exchanged for the HAT domain of either P/CAF or GCN5, other genes seem to be upregulated than with the CBP HAT domain, since different (less differentiated) cell surface markers are expressed (20). Thus, this indicates that the various HATs each have their own substrates and/or that depending on the chromatin context different functional effects are induced.

Whereas writing activating marks seems to be effective, removal of repressive marks could also be of interest for activation of genes. In fact, although (tri)methylation marks were long thought to be relatively stable, various enzymes have now been described to actively remove these methylation marks (124,125). To actively remove the repressive H3K27me3 mark, UTX (126,127) and JMJD3 (128) could be explored in targeting studies, but to the best of our knowledge no such studies have been reported to investigate the effect of removal of this particular mark. However, other histone demethylases, demethylating H3K9 and/or H3K27me2 (JMJD2D or KIAA1718) have been studied in a targeted fashion and removal of the marks was indeed demonstrated. Targeting of JMJD2D to methylated endogenous genes by fusion to the MBD of MeCP2 resulted in the intended reduction in H3K9me3 at the analysed MLH1 gene, while no changes in H3K9me2 or DNA methylation levels were observed (17). Upon analysing the effect on gene expression for this target gene, it appeared that demethylation of H3K9me3 was not enough to induce gene expression (17). Likewise, for another target gene that was assessed (GSTP1), no induction of gene expression could be shown. Molecular marks were not analysed for this gene. The lack of upregulation despite the change in H3K9 methylation might be explained by the fact that no other (assessed) marks changed. In contrast, targeted KIAA1718 (which did cause upregulation of gene expression) showed an increase of H3 acetylation levels, in addition to the expected decreased level of H3K9me2 (111).

Another example of additional histone modifications changing was observed when targeting drosophila Ash1. Not only H3K4me2 levels decreased, H3K9me2 and H4K20me2 marks were increased at a stably integrated reporter gene in *Drosophila* S2 cells (109). However, the induction of H3K9me2 and H4K20me2 is a known function of dAsh1 in addition to the H3K4 methylation. Interestingly, despite the additional induction of the two marks that are associated with gene inactivity (H3K9/H4K20 methylation), upregulation of the reporter gene expression was still observed. The human homolog of Ash1 was not able to change gene expression upon targeting (60). Nevertheless, this is in line with findings that human Ash1 does not methylate H3K4, but can only mono- and di-methylate H3K36, which might be insufficient for GEM (129).

As becomes clear from Table 3, only a few studies addressed the effect of targeting the ‘activating’ enzymes both on modulation of the histone marks as well as on gene expression, whereas others were not intended to assess both. Studies investigating both of these features can give some insights on whether the cause of activation is the induction of the anticipated mark or merely recruitment of other regulatory proteins (6,7). From the studies reported so far, indications can be distilled that the anticipated change in histone modification by targeting of p300 (31), Ash1 (109), Dot1L (19), JMJD2D (17) and KIAA1718 (111) indeed led to increase in gene expression of the targeted gene. However, only studies including the targeting of a catalytic inactive counterpart of the domain can firmly indicate a causative relationship between mark and expression regulation and such studies have been performed for DNA methyltransferases (55–57), Meisetz (108), dAsh1 (109), vSet1 (82), Suv39H1 (29) and G9a (78) as will be described below.

## TOWARD *EFFICIENT* TARGETED GENE EXPRESSION MODULATION

From the above, one can conclude that targeting epigenetic enzymes is a feasible approach to determine functional domains within epigenetic enzymes and to investigate the effect of edited epigenetic marks. Various reports also demonstrate that targeting epigenetic writers or erasers indeed affect gene expression levels, and some studies touched upon the chromatin context requirements. So far, three papers on gene-specific *EGE* have been published, describing the targeting of an epigenetic writer to an endogenous locus through fusion to a gene-specific DBD (29,57,95). Indeed, in these studies gene expression was affected, with some indication of spreading of the H3K9me2 mark (29) or mitotic stability of targeted DNA methylation (57). Despite successful attempts on rewriting epigenetic signatures to modulate gene expression, the studies summarized above indicate that many issues remain to be clarified for this approach to become robust. In this respect, questions to be addressed include: (i) which epigenetic mark or combination of marks needs to be induced/removed in order to efficiently interfere with gene expression (given a particular chromatin context); (ii) is the edited mark mitotically stable or will the native epigenetic marks be restored upon removal of the editor; and (iii) what is the influence of the chromatin landscape in determining the outcome. Depending on the envisioned epigenetic change, the most optimal effector domains need to be engineered to selectively, yet efficiently, execute its activity specifically at the targeted site. Some of the studies summarized by us (Tables 1–3) did address such efficiency and specificity issues in more depth and will be discussed below.

### Direct gene expression modulation

It is subject of a hot debate whether epigenetic marks are the drivers of gene expression regulation or merely associated with expression status (6–8). Indeed, for some enzymes, targeting studies have shown that introducing mutations in the CD or removal of this domain has little or no effect on the induction of changes in gene expression compared with the effect of their larger or full-length counterpart proteins. This indicates that not in all cases catalytic activity of the targeted enzyme is important for an effect on gene expression. Obviously, different studies target the effectors to different chromatin and cellular contexts, and this context will significantly influence the outcome (as described for HATs below). Moreover, differences might also be explained by a variation in expression levels or nuclear entry of the constructs, but this was not addressed in most studies. Despite the fact that it is difficult to distill general rules from the limited amount of studies done so far, some evidence exists that, in particular cases, induction of the mark itself is likely to be sufficient to initiate gene expression differences.

In this respect, targeting of the CDs of Dnmt3a (murine: aa 598-908, human: aa 592-909) and Dnmt3b (murine: aa 557-859) was sufficient to cause targeted DNA methylation and repression of gene expression, whereas targeting of catalytically dead mutants had no effect on expression levels (55–57). Also with respect to H3K4me3, replacing a Glycine residue with an Alanine residue at aa position 278 in the catalytic PR/SET domain (aa 246-365) completely abolished the activating potential of Gal4-targeted Meisetz (108). Similarly, mutating the HMTase domain (E1357K or N1458I) of drosophila Ash1 prevented the increase of H3K4 methylation at the target site, and subsequently eradicated the activation of gene expression, as opposed to the wild-type enzyme (109). With respect to the repressive mark H3K27me3, creating a catalytic mutant by changing one aa of the Set domain of the H3K27 MTase vSet (Y105A or Y105F) was sufficient to prevent the decrease in reporter gene expression which was observed upon targeting the wild-type enzyme (82). Also for SUV39H1, the repressive activity of a construct—where the N-terminal 76 aa of the enzyme were deleted—was completely abolished by mutating the catalytic activity (29). In case of Gal4-G9a, a ΔSet construct could not induce H3K9 methylation and subsequently had no effect on gene expression (78). Vice versa, targeting truncated constructs *including* the HMT activity (aa 210-1202 (78)) of the HMTase G9a and even a domain as small as aa 829-1210 (29) were able to induce H3K9 methylation, and both targeted constructs could efficiently downregulate target gene expression. In general, these studies provide strong indications that epigenetic marks can be instructive in gene expression regulation.

### Indirect gene expression modulation

Next to enzymes which seem to rely solely on their catalytic activity for GEM, other enzymes have been described to possess efficient gene expression modulating activity despite absence of (or mutations in) the CDs. For example for Dot1, the H3K79 MTase from yeast, two mechanisms of derepression can be elucidated, a CD-dependent and a CD-independent mechanism of action. The CD-dependent mechanism was confirmed by targeting of only a small part (aa 172-582: including the HMT domain) of Dot1, which was still able to derepress a target gene integrated in the yeast genome, with similar efficiency as full-length Dot1 (110). On the other hand, targeting of only the N-terminal part (1-237 aa, not including the HMT domain) of Dot1 also induced derepression of gene expression, thus via a CD-independent mechanism. Whereas the CD-dependent mechanism works via reducing the binding of Sir proteins (yeast homologs of the mammalian sirtuin HDAC proteins) to the target through methylation of H3K79, the recruitment of the HAT Gcn5 appears to be required for the CD-independent mechanism. In addition, the N-terminal domain seems to function in positioning the gene away from the nuclear envelope (where heterochromatin normally localizes). Similar effects were observed with hDot1L for which several domains were targeted (aa 1-340, 1-430 and 318-430) using the same model system (110). Although aa 1-430 shows the best effect (better than 1-340), aa 318-430 on its own has no effect. Interestingly, when a truncated part of hDot1L (aa 1-670, including the HMT domain) was investigated in a fusion to MLL (130), less efficient induction of H3K79 methylation was observed when compared with MLL-AF10, which supposedly recruits the full-length endogenous Dot1L, confirming that other domains of Dot1L are required for full activity. Therefore, these reports provide an example of an effector domain containing functional domains apart from the CD which also play a role in achieving GEM.

Similarly, mutating the active site of SirT1 did not abolish all repressive activity. Moreover, deletion of the N-terminal 268 aa (which does not affect the HDAC domain) diminished the repressive effect of SirT1, compared with the full-length enzyme (75). This observation might be explained by the fact that the missing part was shown to recruit histone H1b, which has been associated with heterochromatin. Likewise, a fusion of TetR to a catalytically inactive mutant of LSD1 (K661A) was still active, resulting in reduction of H3K4me2 levels at the target site, be it after longer exposure of the target site to the mutant enzyme than for the wild type (77). The authors suggested that the observed decrease in H3K4me2 levels was a secondary effect due to induced repression of transcription; recruited repressor proteins might in turn recruit the wild-type LSD1. Also for Set2, the histone MTase activity can only explain part of the repression, since two mutations (R195G and C201A) in the CD of this H3K36 MTase both did not abolish repression completely (73). It might be that a substantial part of repression by Set2 lacking the active Set domain is caused by the remaining ability to recruit HDACs, as an association was reported for another H3K36me2 MTase, Smyd2, with HDAC1 and Sin3A (83).

Despite the indications described above that catalytic activity is (at least partially) responsible for the observed effects on gene expression, examples exist of efficient GEM by targeting enzymes *without* CDs and/or catalytic activity. Illustrative here is an example of Dnmt3a: although the CD is known to result in DNA methylation and gene repression, the targeting of the full-length murine Dnmt3a to a reporter plasmid did not result in DNA methylation (30). Despite this lack of detectable induced DNA methylation, repression of reporter gene expression was observed and, as demonstrated by ChIP, proteins with repressive functions, including Setdb1 and HDAC1, were recruited to the promoter site. Thus, in this case, it seems that silencing of the reporter gene was not caused by DNA methylation through mDnmt3a, but indirectly by recruitment of other repressive proteins. Since the observed repressive complex was also formed on endogenous promoters of hypermethylated genes, as demonstrated by ChIP–reChIP, this study underlines the power of targeting mammalian proteins to initiate a natural repressive cascade in the mammalian context.

Regardless of the indirect effect on gene expression, the editing of a mark might be an absolute requirement in order for the intervention to be mitotically stable. This is exemplified by a targeting study in which an Ezh2-mutant lacking its catalytic Set domain still efficiently repressed transcription to 10% of the control level (81). As the Gal4-Ezh2 ΔSet fusion protein was not associated with an increase in H3K27me3 levels at the target site, the repression by the ΔSet mutant of Ezh2 might be caused by sterical hindrance through recruitment of binding partners of the Polycomb repressive complex (PRC2) such as EED and Suz12. Confirming this hypothesis, targeting EED to the reporter, using the same system, resulted in gene silencing to 30% of the control level. Moreover, the repressive effect is no longer observed upon clearance of the inducible expression of Gal4-Ezh2 ΔSet. This transient effect is in striking contrast to the effect obtained by targeting wild-type Ezh2 which showed both induction of H3K27 methylation and prolonged repression of reporter gene expression. Most likely, the introduction of H3K27me3 and the subsequent cascade of events, not achieved by the Gal4-Ezh2 ΔSet fusion protein, are essential for obtaining sustained repression.

Theoretically, the presence of certain (recruiting) domains within the effector domain can decrease the efficiency of the targeted enzyme by causing the effector domain to be captured by endogenous proteins before reaching the intended target site. This was suggested to explain the failure to repress endogenous reporter gene expression by targeting full-length H3K9 MTase Suv39H1 in fusion to a ZF (29). The intact HP1 interaction domain might be captured by endogenous HP1 proteins in heterochromatin, thereby preventing the fusion protein from reaching its target. Deletion of the N-terminal (HP1 interaction domain containing) 76 aa or 149 aa did result in efficient histone methylation and gene repression of a gene in the chromatin context in a ZF-targeting study (29). Even though the deletion construct could not recruit HP1 directly because it lacked the HP1 interaction domain, H3K9me2 was observed as far as 1 kb upstream of the ZF binding site, which suggests that some spreading did occur. This spreading was mediated by the enzymes recruited by the induced mark itself and not indirectly through recruitment of other proteins by the targeted enzyme, as a catalytic mutant did not demonstrate any enrichment in the methylation marks at this upstream region. Despite the successful repression induced by the truncated Suv39H1, inclusion of parts of the N-terminal domain might help to reinforce the repressive effect as targeting of only this domain (aa 1-195) resulted in equally efficient repression of reporter gene expression compared with targeting of full-length Suv39H1 in another study (79). Apparently, in the latter co-transfection study no capturing effect by endogenous HP1 was observed for Gal4-Suv39H1, as the full length could efficiently repress reporter gene expression. Interestingly, deletion of the N-terminal 213 aa of drosophila SU(VAR)3-9 (of which the first 155 aa are lacking in the human homolog Suv39H1) rendered the Gal4 fusion protein ineffective in a co-transfection study compared with its full length, even though this N-terminal part does not contain the Set domain (80). For the full-length enzyme, the strong repressive effect was severely reduced after addition of an HDAC inhibitor, which confirms a role for the interaction of the N-terminal domain with the HDAC RPD3.

**Figure 2.**
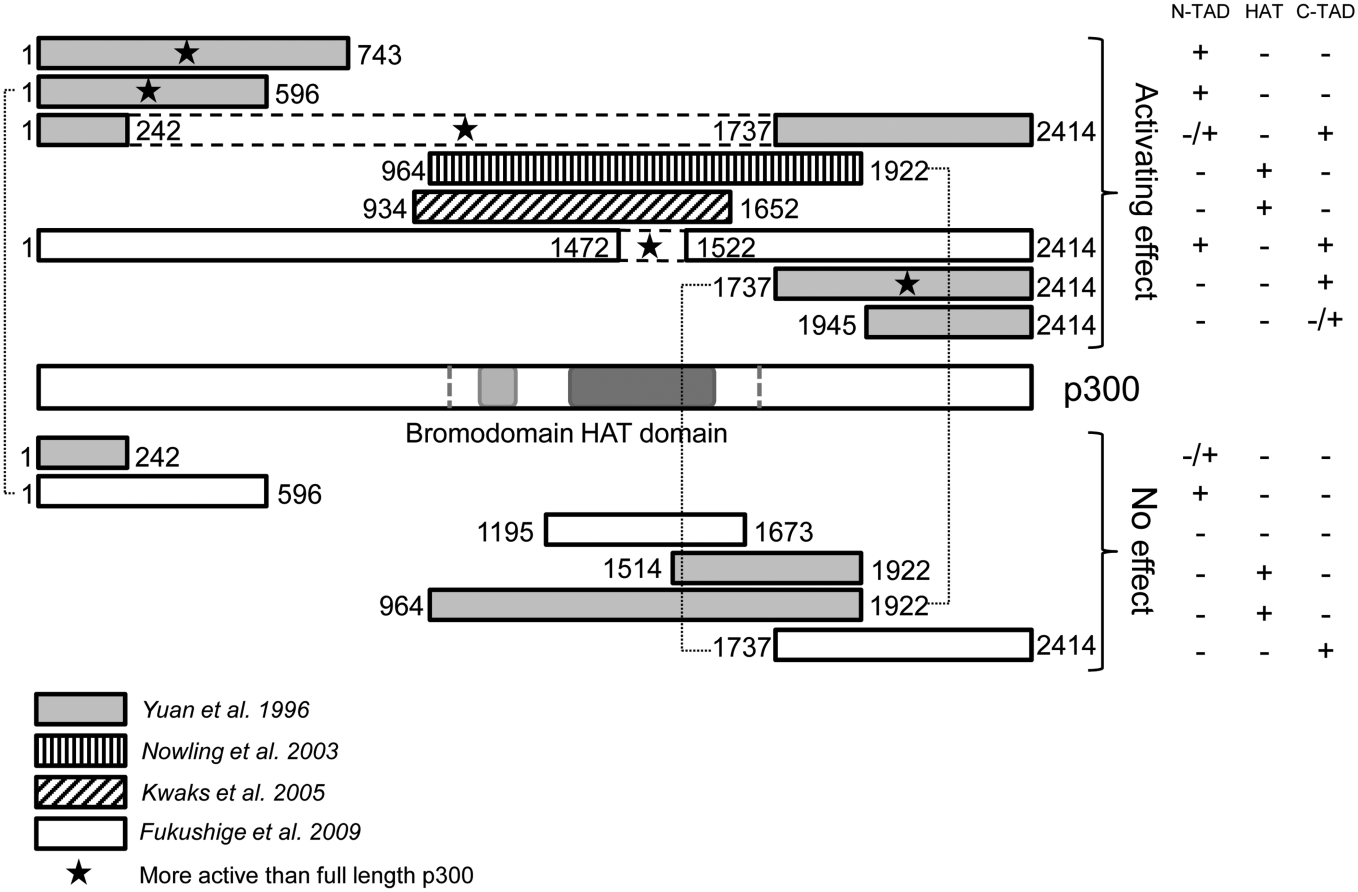
Targeted gene expression regulation capacity by various domains of p300. This figure gives an overview of the domains of p300 that were targeted by fusion to a sequence-specific DBD. Full-length protein actively upregulated the expression of the target gene in all targeting studies reviewed here. The activating domains are shown above and the non-activating domains below the full-length protein. The first and second indicated domain, respectively, indicate the bromodomain and the HAT domain of p300 according to a conserved domain search on the NCBI website. Dashes in the full-length protein show the position of the commercially available ‘HAT domain’ of p300 (Millipore). Numbers indicate aa positions. Black-dashed lines represent a part of the enzyme deleted in the middle of a protein. References are indicated by the pattern of stripes or color within the domain box as explained in the legend. The table at the right side indicates whether the N-terminal activation domain, HAT domain and/or C-terminal activation domain are present in the related construct. Domains indicated with a star shape were more active than the full-length enzyme upon comparison within one study. Thin dotted lines connect two equal domains with different outcomes.

The most extensively studied protein with respect to effects of different domains is the HAT p300 (Figure 2), where at least three domains seem to influence gene expression levels. Targeting aa 964-1922 of p300, which includes the HAT domain, by fusion to Gal4 is sufficient to result in activation of reporter gene expression in one study (98), but not in another (99). Also, targeting of a similar but somewhat smaller HAT-containing domain of p300 failed to induce endogenous target gene expression in an MBD targeting study (18). Interestingly, in these latter two studies, p300 fusion constructs without the HAT domain (Δ242-1737 (99) and Δ1472-1522 (18)) could induce activation of gene expression, even to a higher extent than the full-length protein. These p300 deletion constructs both contain the N-terminal and the C-terminal activation domains, known to form ‘enhanceosomes’, which might explain the observed effects. Indeed, targeting of only the N-terminal (aa 1-596, but not aa 1-242) or the C-terminal domain (aa 1737-2414) induced expression, again outperforming the full-length fusion construct (99). Differences between effects of similar domains in different studies, however, are observed and might be explained by the location of the targeted sites (as will be discussed in the part *‘TOWARD* SPECIFIC *GENE EXPRESSION MODULATION—The DNA binding domain’*). In this respect, in another study, targeting of only the activating N-terminal 1-596 domain, by fusing it to the MBD of MeCP2, could not induce expression of endogenous methylated genes (18). Of course there are many other factors in addition to the location of the targeted site that are likely to play a role in the observed differences in effects caused by similar domains of the p300 protein. For example, the differences in cell lines and constructs (in particular the DBDs) could determine the controversial outcomes. However, within every study some domains of the p300 protein did show an activating effect, indicating that the experimental set-up was available for effective GEM to occur by targeted HATs.

Also for CBP, targeting of only the HAT domain and the CBP2 domain (aa 1099-1877) results in gene activation to a higher extent than the full-length enzyme (102). Deletion of aa 1458-1475 within the HAT domain eliminated the activating effect of the HAT, even when the CBP2 domain is present. The removal of the CBP2 domain—while leaving the HAT domain intact—resulted in a slightly decreased activating potential. This decrease in activating potential might be explained by the CBP2 domain containing a number of binding sites for co-activators, although targeting of only the CBP2 domain did not result in an activating effect (102).

In conclusion, for some epigenetic enzymes GEM is caused through the direct writing or erasing of epigenetic marks, whereas for others recruitment of co-activators or repressors determines functional outcome. However, to achieve efficient, sustained effects (mitotic heritability), the actual editing of epigenetic marks seems warranted.

## TOWARD *SPECIFIC* GENE EXPRESSION MODULATION

### The effector domain

Thus, targeting epigenetic enzymes or domains thereof, can induce efficient modulation of epigenetic marks resulting in GEM. Of utmost importance for general applicability of the approach is the locus specificity of the targeted approach. As exemplified by Gal4-targeted *Drosophila* Ash1, the targeting of domains does not necessarily ensure site-specific effects. The natural Ash1 target gene Ubx, which normally is not expressed in the experimental model used, was re-expressed as well in addition to the intended targeted reporter gene (109).

The re-expression of Ubx upon expression of Gal4-Ash1 was accompanied by induction of H3K4, H3K9 and H4K20 methylation at this endogenous site as also observed for the intended integrated target site. Apparently, the Gal4 DBD was not strong enough to prevent binding of Ash1 to the Ubx gene. In this line of reasoning, it is also important to realize that certain epigenetic enzymes, such as HATs and HDACs were shown to have an effect on non-histone proteins such as transcription factors (131,132), which might influence the cell biological outcome.

Similarly, targeting of DNA methyltransferase M.HhaI and M.HpaII fused to a four-finger ZF resulted in methylation of their coding plasmids in bacteria although these were devoid of ZF binding sites (52). In addition, background methylation was reported for M.SssI (48,49). Contrastingly, the enzyme only efficiently functioned on naked DNA when tethered to the DNA; efficient methylation was observed for ZF-M.SssI for oligonucleotides containing the ZF binding site, but not for oligonucleotides without the ZF binding site (48). Although the affinity of M.SssI itself for DNA was decreased upon fusing the enzyme to ZFs, it still seems to be too high to allow its site of action to be restricted by DBDs (48). Namely, similar methylation efficiencies were observed for targeted versus untargeted M.SssI in yeast cells, for a non-ZF target locus as well as for the targeted locus (49). Such off-target effects underline the need for MTases strictly functioning at the predetermined target.

A promising way to diminish off-target effects of epigenetic enzymes in targeted fusion proteins is to engineer less active mutants of the enzyme to be fused to the DBD. In this respect, we constructed the M.SssI mutant C141S, with a remaining activity of <5% of the wild-type activity and conjugated this mutant to a gene-targeting TFO (50). No other CpGs than two targeted CpGs were efficiently methylated in a region of 700 bp of the promoter or in an amplicon of 400 bp investigated within the reporter gene of the plasmid upon co-incubation in a cell-free system, confirming locus-specific DNA methylation. Likewise, diverse mutants of M.HhaI and M.HpaII have been constructed (52). For the mutant M.HhaI^Q237G^, which has a remaining methylation activity of less than 5% in *in vitro* enzyme assays, target-specific methylation was confirmed by absence of restriction of the coding plasmid, including the target site, by methylation sensitive restriction enzymes. For M.HpaII, an F35H mutant with reduced activity was created of which the mutated aa normally aids in positioning the adenine ring of *S*-adenosylmethionine (SAM) in the protein binding pocket (52). Also this mutant shows target-specific methylation in the same system. Evidently, the same approach of creating lower activity/affinity mutants could be used to restrict activity of histone modifying enzymes to the intended loci.

As an alternative to constructing mutant enzymes, the so-called ‘split enzyme approach’ has been explored to improve the specificity of targeted methylation. Only when the split parts of the protein localize at neighboring sites, the parts of the enzyme can combine and methylate the target sequence. For M.HhaI, a plasmid encoding two separate three-finger ZFs fused to complementary halves of the enzyme and also containing the ZF target sites was transformed into *Escherichia coli* (53). Site-specific methylation of the cytosine flanked by the two ZF binding sites was confirmed by bisulfite sequencing. A recent study demonstrated the phenomenon of fragment complementation for the CG-specific methyltransferase M.SssI, which may open the way for applying the split enzyme approach for targeting any CpG site without sequence context limitation (133). Although selectivity was not fully supported in another M.HhaI split-enzyme study (134), the approach of splitting enzymes (as reviewed in reference (135)) might provide a promising tactic for increasing specificity of *EGE*.

Despite off-target effects, several examples exist where the effect of the targeted enzyme seems to be restricted, since it only takes place when indeed the recognition site of the DBD is present. For example, when targeting human Dnmt3a CD to mitochondrial DNA, no off-target methylation was shown for two distant mitochondrial regions, suggesting usefulness of human Dnmt3a in targeting purposes (56). Likewise, expression of Gal4-CBP had no effect without Gal4 targets being present in the reporter plasmid in a cotransfection study (102).

### The DNA binding domain

Off-target effects, on the same chromosome and on other chromosomes, might also be envisioned to occur due to flexible linkers between the DBD and effector domain in combination with dynamic movement of chromatin. Despite a specific binding of the DBD to its unique genomic target site, the effector domain might get in contact with distal sequences due to chromatin folding, but also due to cis and trans interchromosomal interactions. The basic reach area of epigenetic enzymes has been investigated using oligonucleotides containing DBD recognition sites. In this respect, using oligonucleotides with varying distances (2–32 nt) between the ZF binding site and the target CpG, methylation induced by Zif268-M.SssI was shown to occur preferentially at cytosines 16 or 22 bp upstream of the ZF binding site (48). Although this preference likely reflects the length of the linker between ZF and DNA MTase (19 aa), linker dependence was not further investigated in this study. As for Zif268-M.SssI, the flexibility of the ZF-M.HpaII fusion protein was tested *in vitro* and appeared to be limited, again likely dependent on its linker length (21 aa) (51). A distance of 10–40 bps between the ZF target site and the M.HpaII recognition site was most successful for binding and methylation activity of the fusion protein, with optimums at 13 and 34 bps. At 16 and 17 bps distance, a weaker point was detected, which might indicate a position unable to be reached by the effector part of the fusion protein.

Based on the above, the reach area of a ZF-DNA MTase fusion in cell-free systems is limited. Although cellular experiments suggest that recruitment of spreading mechanisms can easily enhance the initial effect, it is of importance to ensure the first hit is efficient. In that respect, it is necessary to know in which direction the effect will have to take place, upstream or downstream of the DBD recognition site, as the site of effect might be determined by the orientation of the effector domain relative to the DBD. For example, for ZF-M.SssI, the direction in which the methylation took place, upstream, was in line with the position of M.SssI in the ZF fusion protein (C-terminal) (48). In another study, for ZF-M.HpaII a preference for methylation of the 3′ end of the target site was observed, which is expected because of the orientation of the ZF-M.HpaII fusion protein on the target DNA (52). Also the observed DNA demethylation upon targeting VP64 was in the expected direction (61). Such orientation dependency, however, might not be observed for all constructs as no clear orientation dependency was observed when targeting a ZF-Dnmt3a CD fusion to an endogenous gene (57).

In addition, most likely it is necessary to modulate more different epigenetic marks to obtain a sustainable effect. As far as known, the effect of targeting two different epigenetic enzymes to the same repeat of DBD recognition sites has not been assessed. However, targeting of vErbA (recruiting the NCoR/SMRT co-repressor complex, executing HDAC activities) and the H3K9 MTase G9a to the same promoter showed an increase of the repressive effect observed compared with targeting either one of the proteins alone (29). Despite that it becomes clear from the above that induction of one epigenetic mark can be sufficient to cause a cascade of events leading to prolonged effects on gene expression, more research into combined targeting of epigenetic writers and/or erasers would be beneficial, especially within the endogenous chromatin context.

Depending on the epigenetic enzyme, it is likely that the genomic site where the effector domain is targeted to (e.g. relative to the transcription start site) is of importance in determining the functional outcome. The target site position at least seems to be playing a role in the case of HATs, as this might explain the contradiction between the two studies targeting p300 aa 964-1922. In one of the studies, the Gal4 binding sites were situated upstream (99) whereas in the other the sites were situated downstream (98) of the transcription start site. Only in the study where the Gal4 binding sites were situated downstream of the transcription start site, an activating effect was seen, as also shown for full-length p300 in a different study (100). Fusion of Gal4 to P/CAF again only resulted in upregulation of gene expression after cotransfections when the Gal4 binding sites were situated downstream, not upstream, of the transcription start site of the reporter gene. When the Gal4 binding sites were located upstream, close to the TATA box, an SP1 binding site was needed for reporter gene activation by targeted P/CAF (100).

Evidently, not only the characteristics of the fusion protein, comprising the DBD, the linker and the epigenetic enzyme, influence the specificity and efficacy of the effect. Rational selection of the target site itself might also be beneficial. In this respect, native chromatin context requirements might be identified in the future which allow efficient *EGE*. From the studies described in this review, it becomes clear that one target site can be sufficient, underlining the feasibility of targeting epigenetic enzymes using a single gene-specific DBD. Actually, a repeat of target sites is not per se required to improve the effect of histone modifying enzymes, as similar repression has been shown by LexA-RPD3 when the reporter plasmid contained just one LexA binding site when compared to a reporter plasmid with four LexA binding sites (73). For Gal4-Suv39H1, adding an additional Gal4 binding site to the reporter plasmid did not improve the repressive effect either (79).

## CONCLUSION

Apart from the ultimate goals of inducing efficient and permanent GEM, *EGE* is likely to provide valuable insights in cause versus consequence of epigenetic marks with respect to GEM. It is still a matter of debate whether DNA methylation and post-translational histone modifications influence the gene expression levels directly or if they are merely byproducts of transcription (6,7,136)*.* For example, DNA demethylation was reported to be associated with active histone marks in post-mitotic cells, but not with transcriptional activity (137). Targeting minimal CDs of epigenetic writers (and their catalytically dead mutants) to defined chromatin environments allows comparisons determining the effect of the edited mark on higher order chromatin and on gene expression. In this respect, *EGE* might provide a unique tool to eventually settle this cause versus consequence debate.

Before *EGE* can become a straightforward approach, however, the influence of the chromatin context on the dynamics of epigenetics has to be addressed in a systematic manner. It is expected that the positioning of the effector domain as well as the promoter type might affect the ultimate outcome. Similarly, dependent on the cell type, different regulatory protein complexes might be recruited to the same epigenetic mark (138), and different histone (variant) turnover rates and clipping of the histone tails will determine the transient versus mitotically stable nature of the induced mark (139). In this context, Verschure *et al.* (manuscript in preparation) designed a coarse-grained stochastic model systematically adding epigenetic regulatory levels of increased complexity (i.e. epigenetic enzyme binding, its spreading, subsequent recruitment of regulatory proteins and chromatin folding) allowing to simulate and interpret the mechanistic and dynamic behavior of a nucleosomal stretch to attain a defined epigenetic composition.

Initial publications showed the promise of *EGE* for a handful of genes [MASPIN, Sox2, VEGF-A (29,57,95)] and from these studies some indications on heritability (DNA methylation) and spreading (H3K9me) can be distilled. Importantly, these proof-of-concept studies on targeted methylation clearly show the intended repressive effect on gene expression. Although it might be more challenging to effectively compete with spreading mechanisms to overwrite repressive histone marks, accessibility of inactive chromatin presents no limitations as ATFs have been successful in re-expressing silenced and even imprinted genes (61,140). With recent developments in the targeted DNA demethylation field (21), combinations of erasers together with certain writers might prove potent in this respect. In fact, *EGE* designed for upregulation of a gene of interest, is advantageous over cDNA approaches as with *EGE* all isoforms can be produced in their natural ratios and expression levels are controlled from the natural promoter and through natural signaling pathways. In addition, silencing via *EGE* through appropriate combinations of marks might prove to be advantageous over approaches like siRNA because the effect would be sustained after clearance of the drug (hit and run approach) (57), without saturating/affecting other cellular (RNAi) processes (39).

With respect to such other gene-specific gene expression modulating approaches (gene therapy, RNA interference), *EGE* promises several advantages, including the broad spectrum of delivery possibilities, ranging from chemical gene-specific epigenetic inhibitors (141) to direct mRNA (28) or protein delivery (27,142) of the epigenetic editors. Eventually, it will be necessary to achieve efficient, cell- or tissue-specific delivery of the *EGE*-device if to be used as therapeutic agent. Although this requires further research, the use of complexes of antibodies recognizing specific cells or tissue and a cationic lipid or liposomes might be powerful approaches to ensure tissue or cell-type specificity (143).

The need for novel epigenetic therapies is exemplified by the numerous ongoing clinical trials to test inhibitors of epigenetic enzymes (144,145). Although promising, current (FDA approved) epigenetic drugs severely lack specificity not only with respect to the intended target (also unintended non-chromatin proteins are affected), but more importantly with respect to the genome-wide effects (146). The identification of epigenetic marks or combinations of marks which efficiently interfere with gene expression profiles will open up new avenues in biomedical research. As virtually any (undruggable) gene can be targeted for up- and downregulation (22,28), *EGE* adds a novel approach to the biomedical arena to investigate gene functions, to validate therapeutic targets and even to be further optimized to become a (synergistic) therapeutic approach (147).

## FUNDING

National Dutch Scientific Research Organisation [NWO/VIDI/91786373 to M.G.R.; NWO/Meervoud/83607001 to P.J.V.]. Funding for open access charge: National Dutch Scientific Research Organisation [NWO/Open Access/036.001.799 to M.G.R.].

*Conflict of interest statement*. None declared.
